# Supplementary material for: Magnitudes of post-abortion family planning utilization and associated factors among women who seek abortion service in Bahir Dar Town health facilities, Northwest Ethiopia, facility-based cross-sectional study
Source: PLoS One. 2021 Jan 20;16(1):e0244808. doi: 10.1371/journal.pone.0244808 (PMC7817005; doi:10.1371/journal.pone.0244808)
Supplement: Attachment — Submitted filename: renamed_669b2.docx [file pone.0244808.s002.docx]

**Introduction**

Para 2: “Globally an estimated 55.9 million abortions occur each year, from this 49.3 million occur in the developing”. This sentence is obscure, please make it clear.

Para 2: What the author meant by writing “long-term disability”?

Para 3: “Studies conducted in developing countries showed that the prevalence of post abortion family planning utilization was 73%, 73%, 81%, and 97.7% in Africa and Asia, Pakistan, India, and Brazil respectively” . Pakistan and India are Asian countries, why do you mention prevalence in Africa and Asia?

The last paragraph: Why do you conduct the same research in this area since there are studies concerning the utilization of family planning service in your country?

**Method**

The author should provide the data on the health institutions, such as the number of abortions and socioeconomic and obstetric profile of population studied.

I missed one paragraph describing inclusion and exclusion criteria.

It’s not clear the criteria for the inclusion of explanatory variables in the multivariate logistic regression. It should be explained.

**Discussion**

Again, why do you conduct the same research in this area since there are studies concerning the utilization of family planning service in your country? It is important to compare this result with findings from other studies from Ethiopia and eventually from abroad? The author stated” in the number of respondents who counseled about post abortion family planning” when explaining the difference, this is not convincing.

When the author explain: “The odds of post-abortion family planning use among women who received post-abortion family planning counseling were higher than the counterpart”, you should focus on the benefits which post-abortion family planning counseling might bring to abortion patients.

I missed one paragraph explaining: “sex preference of health care providers, and counseling about post-abortion family planning were predictors of post-abortion family planning method utilization”.

**Conclusion**

It should be shorter and concise. Recommendations should be more specific and target the risk factors in your research.
