# Supplementary material for: Magnitudes of post-abortion family planning utilization and associated factors among women who seek abortion service in Bahir Dar Town health facilities, Northwest Ethiopia, facility-based cross-sectional study
Source: PLoS One. 2021 Jan 20;16(1):e0244808. doi: 10.1371/journal.pone.0244808 (PMC7817005; doi:10.1371/journal.pone.0244808)
Supplement: Attachment — Submitted filename: Reviwer Comments.docx [file pone.0244808.s003.docx]

**Reviewer Comments**

The paper is good but is riddled with typos and could do with thorough editing.

A few points and comments mostly for clarification:

1. While the sample size is clear, it is not clear how the clients for the study were recruited and agreed to participate in the study. Were all the 408 women post abortion care clients – please specify in the methodology section? In addition, please clarify the inclusion and exclusion criteria.
2. Under method: Please clarify the number of health facilities as 408 post abortion clients in one month seems to be high.
3. Please clarify whether these women had come for abortion services, is it legal or whether they had come for treatment of abortion complications.
4. The difference by type of treatment is specified please clarify who the level of the provider (nurse, midwife, doctor etc.) as this has implications on whether the client was counseled or not.
5. Four towns as well as the capital are mentioned as part of the comparison – are these like the study area in terms of whether these are rural, similar client demographics and socio-cultural factors that affect family planning decision making? In other words, did they have the same variables that influence FP uptake.
6. Difficult to understand what was evidence this study added to what is already known to influence PAFP from Ethiopia itself as well as global.
