# Supplementary material for: Magnitudes of post-abortion family planning utilization and associated factors among women who seek abortion service in Bahir Dar Town health facilities, Northwest Ethiopia, facility-based cross-sectional study
Source: PLoS One. 2021 Jan 20;16(1):e0244808. doi: 10.1371/journal.pone.0244808 (PMC7817005; doi:10.1371/journal.pone.0244808)
Supplement: Attachment — Submitted filename: Response to reviwers.docx [file pone.0244808.s004.docx]

Point by point responses

|  | **Reviewer Comments** |  |
| --- | --- | --- |
|  | **General comments** | **Authors response** |
|  | The paper is good but is riddled with typos and could do with thorough editing. | Thank you for your suggestion. For addressing the issue we consulted senior public health staff and language professors in my university. |
| **1** | While the sample size is clear, it is not clear how the clients for the study were recruited and agreed to participate in the study. Were all the 408 women post abortion care clients – please specify in the methodology section? In addition, please clarify the inclusion and exclusion criteria. | Thank you for your comment. Based on your comment we amended it on the revision manuscript.  Line number 189-191 |
| **2** | Under method: Please clarify the number of health facilities as 408 post abortion clients in one month seems to be high. | Thank you for your comment. Based on your recommendation we add the numbers health facilities included in the study on the revision manuscript.  Line number 198-199 |
| **3** | Please clarify whether these women had come for abortion services, is it legal or whether they had come for treatment of abortion complications. | Thank you for your comment. All selected facilities provided legal abortion services based on the country law and post abortion care services for those women who came for emergency post abortion care services. We included this information in method section.  Line Number 199-201 |
| **4** | The difference by type of treatment is specified please clarify who the level of the provider (nurse, midwife, doctor etc.) as this has implications on whether the client was counseled or not. | Thank you for your comment. In Ethiopia there is special training (post abortion care training) gave for the profession who gave abortion service otherwise they are not eligible for providing abortion services whatever the level and the types of health professional. The main aim of the training is to increase the capacity of the professionals to provide all components of abortion care including counseling and providing post abortion family planning care. Otherwise there is no variation on counseling’s of family planning on the level of the provider (nurse, midwife, doctor etc.) |
| **5** | Four towns as well as the capital are mentioned as part of the comparison – are these like the study area in terms of whether these are rural, similar client demographics and socio-cultural factors that affect family planning decision making? In other words, did they have the same variables that influence FP uptake? | Thank you for your comment. The socio-cultures of the capital (Addis Ababa) is almost the same in the study area Bahir Dar with regards of the availability of facilities like health facilities, educational centers that directly influence the uptakes of family planning services since Bahir Dar is one of the second economic centers of the country. |
| **6** | Difficult to understand what was evidence this study added to what is already known to influence PAFP from Ethiopia itself as well as global. | Thank you for your comments. As a country Ethiopia implements a strategy that focus on providing post abortion family planning services for all women who receive abortion services while this study find out, the current levels of the post abortion family planning in study area was low only half of them were used family planning services that is contradict the current implemented strategy. Therefore, the lesson we learned from this study is still the country needs to do great attention in the area. In addition, providing counseling for clients is one of the means to improving the uptakes of post-abortion family planning. Therefore, this finding providing a clue to see the way the strategy was implemented. |
|  | | |
| **Additional points** | | |
|  | **Introduction** |  |
| **1** | Para 2: “Globally an estimated 55.9 million abortions occur each year; from this 49.3 million occur in the developing”. This sentence is obscure, please make it clear. | Thank you for your comments. Based on your comment we correct it on the updated manuscript.  Line number 8 to 9 |
| **2** | Para 2: What the author meant by writing “long-term disability”? | Thank you for your comments. As we know abortion responsible for maternal mortality and morbidity. From the morbidity abortion causes “long-term disability.” Long term disability mean scarring of the uterine lining, secondary infertility; ectopic pregnancy, damage to internal organs and breast cancer. |
| **3** | Para 3: “Studies conducted in developing countries showed that the prevalence of post abortion family planning utilization was 73%, 73%, 81%, and 97.7% in Africa and Asia, Pakistan, India, and Brazil respectively” . Pakistan and India are Asian countries, why do you mention prevalence in Africa and Asia? | Thank you for your comment. There is no specific reason that we include African and Asia. But we are interested to show what the magnitudes post-abortion family planning utilization out sides of the study country because of that we found astudy conducted in eight country found in Africa and Asia (Benson J, Andersen K, Brahmi D, Healy J, Mark A, Ajode A, et al. What contraception do women use after abortion? An analysis of 319,385 cases from eight countries. Global public health. 2018;13(1):35-50.) that is why we include it. |
| **4** | The last paragraph: Why do you conduct the same research in this area since there are studies concerning the utilization of family planning service in your country? | Thank you for your comment. based on your comment we incorporate some additional evidence that lead as to conduct this study by including “The governments of Ethiopia implemented the new health sector transformation plan to improve the health system utilization one of the focus areas is expanding the infrastructure and post abortion family planning services since 2015/26.”  Line number 178 to 183 |
|  | **Method** |  |
| **5** | The author should provide the data on the health institutions, such as the number of abortions and socioeconomic and obstetric profile of population studied. I missed one paragraph describing inclusion and exclusion criteria.  It’s not clear the criteria for the inclusion of explanatory variables in the multivariate logistic regression. It should be explained. | Thank you for your comment. Based on your comment we amended it on the revision manuscript by including the inclusion criteria “All women who came for abortion care services during the study period were included in this study while women who critically ill and any sign of infection were not included in this study.”  Line number 189-191 |
|  | **Discussion** |  |
| **6** | Again, why do you conduct the same research in this area since there are studies concerning the utilization of family planning service in your country? It is important to compare this result with findings from other studies from Ethiopia and eventually from abroad? The author stated” in the number of respondents who counseled about post abortion family planning” when explaining the difference, this is not convincing. | Thank you for your comment. There is different research conducted in Ethiopia in the area of post abortion care before 2015. But in 2015/16 Ethiopia design and implemented the new Health sector transformation plan since with aim of improving quality and equity, universal health coverage and transformation. One of the main focus areas is expanding the infrastructure of post abortion care and components of services. However there is limited finding with area of post abortion family planning services to understand whether there is improvement in the uptake of post abortion family planning. That why we conduct this research. |
| **7** | When the author explains: “The odds of post-abortion family planning use among women who received post-abortion family planning counseling were higher than the counterpart”, you should focus on the benefits which post-abortion family planning counseling might bring to abortion patients. | Thank you for your comment. We incorporate it on the updated manuscript “Counseling is one of the critical elements in the provision of quality family planning services. It is a means, provider’s help clients make and carry out their own choices about reproductive health and family planning.”  Line number 330 to 333 |
| **8** | I missed one paragraph explaining: “sex preference of health care providers, and counseling about post-abortion family planning were predictors of post-abortion family planning method utilization”. | Thank you for your comment. We know sex preference of the professional is one of the significant variables on multivariate analysis. But we are not confortable to discuss the variable since it’s not such important for intervention. |
|  | **Conclusion** |  |
| **9** | It should be shorter and concise. Recommendations should be more specific and target the risk factors in your research. | Thank you for your comment. Based on your comment we correct conclusion and recommendation section on the updated manuscript.  Line Number 346 to 349 |
