# Supplementary material for: Magnitudes of post-abortion family planning utilization and associated factors among women who seek abortion service in Bahir Dar Town health facilities, Northwest Ethiopia, facility-based cross-sectional study
Source: PLoS One. 2021 Jan 20;16(1):e0244808. doi: 10.1371/journal.pone.0244808 (PMC7817005; doi:10.1371/journal.pone.0244808)
Supplement: Attachment — Submitted filename: Response to Reviewers.docx [file pone.0244808.s005.docx]

Response to review

1. There are still major concerns about your article. Notably, a professional copyeditor must be contacted to proof your manuscript for typographical errors and standard English.

*Thank you for your suggestion. For addressing the issue we consulted senior public health staff and language professors in my university. We also used online software specifically grammerly and scribens (check for correctness of spellings).*
